# Bridging bronchus, type six, as a new rare case of a bronchial anomaly

**DOI:** 10.1186/s40981-016-0070-5

**Published:** 2016-12-07

**Authors:** Ashraf El-Molla, Mohamed Daabiss, Rashed Al-Otaibi, Hussein Al-Qudaihy, Samir Bawazir

**Affiliations:** 1 Department of Anesthesia, Ministry of Health, Cairo, Egypt; 2 Department of Anesthesia, Pharos University, Canal El Mahmoudia Street, Alexandria, Egypt; 3 Department of Anesthesia, Director of Anesthesia Department, Prince Sultan Military Medical City, Riyadh, Kingdom of Saudi Arabia; 4 Department of Anesthesia, Prince Sultan Military Medical City, Riyadh, Kingdom of Saudi Arabia; 5 Pediatric Division of Otorhinolaryngology, Head and Neck Surgery, Prince Sultan Military Medical City, Riyadh, Kingdom of Saudi Arabia

**Keywords:** Bridging bronchus, Anomaly, Tracheal stenosis, Anesthesia

## Abstract

In 1976, Gonzales-Crussi et al. (Am. J. Dis. Child. 130:1015–18, 1976) introduced the first case of bridging bronchus as a rare bronchial branching anomaly; since then, only 14 worldwide cases was described. We suggest our case might be number 15 and could be the first case of type six of this bronchial anomaly. We present a case of a 10-month-old infant with bridging bronchus, congenital tracheal stenosis, and double outlet right ventricle who underwent major laparoscopic surgery for repair of gastrointestinal anomalies to raise awareness of this rare underdiagnosed congenital anomaly and a thorough discussion of the tracheobronchial anomalies and its clinical implications.

## Background

Bridging bronchus (BB) is a rare congenital bronchial anomaly [1] that may be associated with a tracheal anomaly such as congenital tracheal stenosis (CTS) [2]. Tracheobronchial anomalies, when associated with congenital heart diseases (CHD) such as double outlet right ventricle (DORV), present a great clinical challenge which needs multidisciplinary teamwork and proper communications among neonatologists, cardiologists, radiologists, otolaryngologists, pediatric surgeons, and anesthetists for proper diagnosis and management [3].

Written informed consent from patient’s parent was obtained before case report publication and available on editors’ request.

## Case presentation

Seven months ago, a 10-week-old male infant was scheduled for a pull-through surgery of the colon and intestine via combined laparoscopic and perineal surgery.

He was delivered at term to a 22-year-old woman by caesarean section due to failure to progress after rupture of membranes. Prenatal ultrasonography was normal except for the heart which could not be properly visualized. During labor, there was abnormal heart rate variability. Apgar scores were 5 and 7 at 1 and 5 min respectively while birth weight was 2.78 kg. There were pansystolic murmur and respiratory distress that required tracheal intubation, resuscitation, and admission to the neonatal intensive care unit (NICU) on mechanical ventilation.

Provisional diagnosis was a complex CHD presented by DORV, transposition of great arteries (TGA), ASD, VSD, aortic coarctation, vertebral anomalies, and VACTERL association. Colostomy was carried out for the imperforate anus.

At 2 weeks old, the baby underwent patch reconstruction of the aortic arch with pulmonary artery banding. During this procedure, he experienced two attacks of intraoperative cardiac arrest due to severe hypoxia and respiratory acidosis. Cardiopulmonary resuscitation was successful. In NICU, the trachea remained intubated on pressure support ventilation (PSV) because of several unsuccessful attempts of weaning from mechanical ventilation and tracheal extubation.

**Fig. 1:**
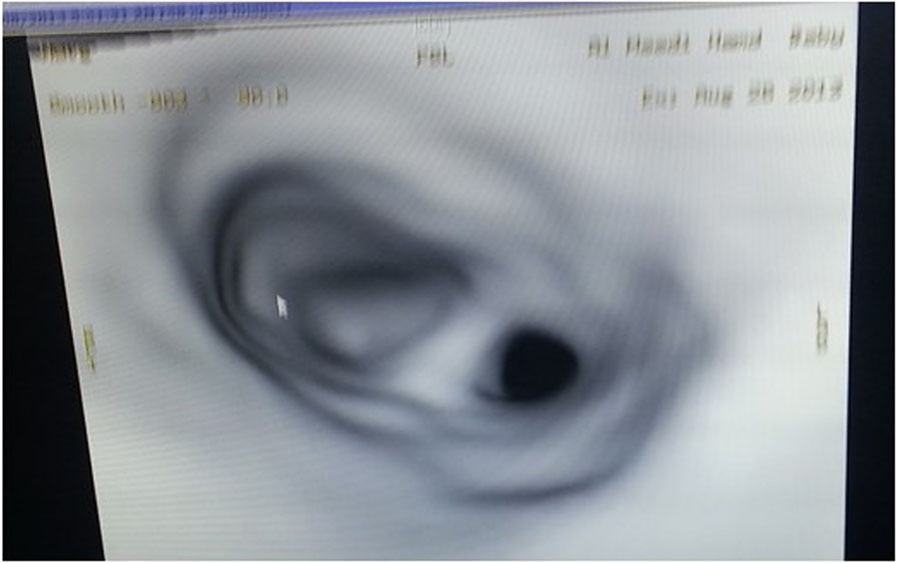
The bronchoscopic view of the distal tracheal stenosis

Two weeks later, microlaryngobronchoscopy was done which revealed a blind distal tracheal pouch and a 2-mm-diameter distal tracheal stenosis, 2 cm above the carina (Fig. 1).

**Fig. 2:**
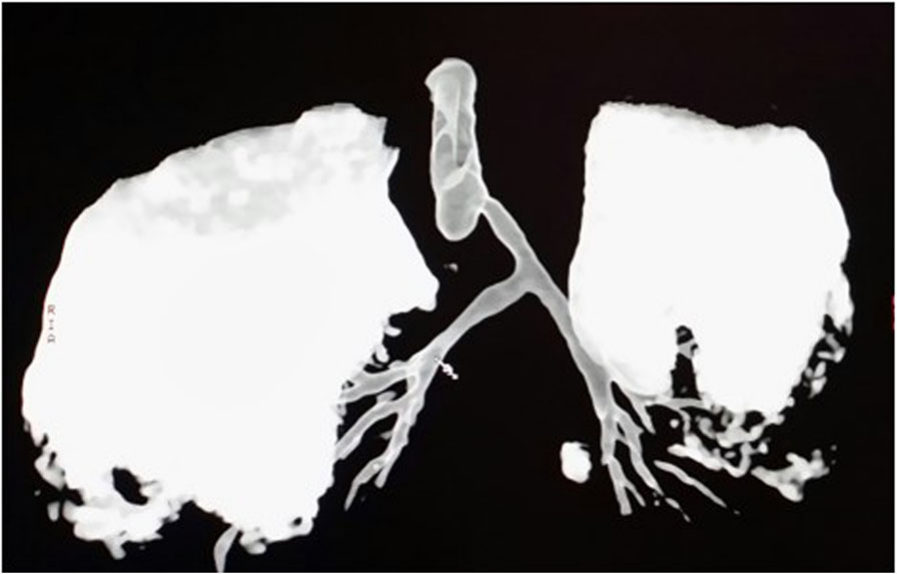
Three-dimensional reconstruction CT of the tracheobronchial tree

Three-dimensional reconstruction CT showed that the left main bronchus (LMB) originated just above the stenosed distal tracheal pouch with left deviation and concentric stenosis of the distal 1 cm of the trachea (Fig. 2).

At the age of 10 months old while his weight was 4.8 kg, the infant had underwent a laparoscopic surgery for repair of the gastrointestinal anomalies; the baby was transferred to the operating room with a 3.5-mm endotracheal tube (ETT) inserted into the trachea. The airway length of the ETT was 9 cm from tip to lips on mechanical ventilation. An esophageal temperature probe, a right internal jugular vein catheter, and right femoral artery catheter were inserted. Intraoperative monitoring included SpO_2_, ECG, invasive blood pressure, and EtCO_2_.

His vital signs were within the limits. Blood pressure (BP) of 80/5 mmHg and the baseline ECG showed sinus rhythm at 122 beats/min. General anesthesia was maintained with sevoflurane and recurring boluses of cisatracurium and fentanyl. Our ventilator strategy was to prolong the inspiratory time to overcome the increased resistance and achieve adequate lung expansion by using pressure-controlled ventilation (PCV) with peak inspiratory pressure of 18–20 cmH_2_O while keeping respiratory rate at 20–25/min to allow adequate time for expiration and to prevent gas trapping to keep ETCO_2_ at 30–40 mmHg while FiO_2_ was 0.5–0.6 to keep SpO_2_ at 95–97% by.

The laparoscopic procedure started with a stable hemodynamics whereas acid–base status revealed mild respiratory acidosis (PaCO_2_ of 50–55 mmHg) while EtCO_2_ was 40–45 mmHg which was improved by an increase of the RR.

After 40 min of positioning baby in Trendelenburg, severe hypercarbia (90–95 mmHg) and hypoxemia (50–60%) were documented associated with a drop in heart rate from 138 to 71/min. The surgeon stopped the laparoscopic procedure. Pneumoperitoneum deflation, restoration of supine position, verification of proper tracheal tube (TT) placement, and proceeding manual ventilation successfully relieved the partial obstruction as indicated by improvement in the vital signs and arterial blood gases. We successfully completed our anesthetic course with our planned ventilator strategy to reduce air trapping beyond the stenosis by decreasing the inspiratory/expiratory ratio to increase the expiratory time and decreasing the respiratory rate to allow CO_2_ wash through the stenosed segment so the pressure-controlled ventilation worked well.

After that, the surgeon resumed the laparoscopic procedure at a 6–8-mmHg insufflation pressure for 70 min with stable hemodynamics. The perineal surgery concluded approximately 2 h afterward. On completion of the whole procedure, the child was transferred back to NICU, trachea intubated on PSV with an uneventful postoperative course.

### Discussion

**Fig. 3:**
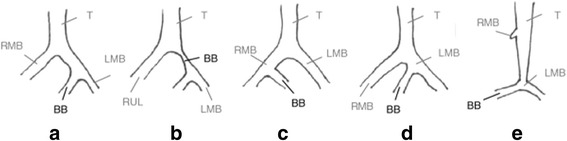
Types of bridging bronchus [16]. (**a**) type 1 Bridging Bronchus (BB). (**b**) type 2 BB. (**c**) type 3 BB. (**d**) type 4 BB. (**e**) type 5 BB

Bridging bronchus is an anomalous bronchus that was grouped into five types. Gonzales-Crussi et al. [1] defined the first type of BB as a large bronchial branch which originates from the left main stem bronchus, bridged the mediastinum from the ipsi- to the contralateral lung, and provides the right lower and middle lobes; but in our case, it provides also the upper lobe (Fig. 3).

Starshark et al. referred to BB associated with several other congenital anomalies [4]. Recognition of BB may be delayed due to misinterpretation of the pseudocarina or tracheal bifurcation. In tracheal bronchus, the origin of the right upper bronchus is at a higher level than a normal bifurcation [5]. It has been stated that clinical differentiation is important because the patients with BB, as in our case, often show tracheal and left main bronchus (LMB) stenosis [6].

The fifth type of BB was described by Medina et al. and Wills et al., where the trachea appeared as carrot shaped and from the distal pseudocarina localized at a lower thoracic vertebral level than the normal position of the carina; the BB arises from the left main bronchus and supplies the right lower and middle lung lobes. The right main bronchus is aborted and represented by a small diverticulum of the trachea, and the right upper lung lobe is absent [2, 6].

Our case presents the sixth type of BB; complete right bronchial agenesis and blind distal tracheal pouch. LMB originated just above the stenosed distal tracheal pouch (Fig. 1). The bridging bronchus takes off from the LMB at the pseudocarina situated at the level of the interspace between the fifth and sixth thoracic vertebral level.

To the best of our knowledge, this is the first report in which BB is associated with complete right main bronchial agenesis and a blind tracheal pouch below the origin of LMB. BB is also supplying the right upper lung lobe, not only middle and lower lobes as in the earlier five types.

We expect that the observed intraoperative hypoxemia and hypercarbia in our case resulted from tracheobronchial stenosis and TT migration to the blind tracheal pouch and hence resulted in a partial airway obstruction as happened in the previous surgical procedure at the age of 2 weeks. This was rapidly corrected by stopping the laparoscopic procedure, by pneumoperitoneum deflation, and by restoration of supine position as well as by manual ventilation which successfully relieved this partial obstruction as indicated by improved oxygen saturation, reducing severe hypercarbia and air trapping ensuring adequate ventilation parameters without the need for intraoperative fiberoptic bronchoscopy.

Congenital heart disease (CHD) is the most common birth defect [7]. DORV patients may have too much or too little pulmonary flow. Our patient has increased pulmonary blood flow and inadequate systemic blood flow secondary to an underdeveloped left ventricle, ascending aorta, and aortic arch. Systemic circulation is provided by blood flow from the pulmonary artery (PA) via the patent ductus arteriosus (PDA) to the aorta, and surgical therapy was directed to the repair of the aortic arch and reducing the increased pulmonary blood flow by placing PA band, which restricts pulmonary blood flow and prevents overwhelming of the pulmonary circulation [8].

Anesthetic manipulations such as hypocapnia and high FiO_2_ should be avoided as they reduce pulmonary vascular resistance and increase the pulmonary blood flow that will steal blood flow from the systemic circulation so hypercapnia is permitted to keep pulmonary vascular resistance (PVR), and the delicate balance between systemic vascular resistance (SVR) and PVR should be maintained, besides to proper control and stabilization of intravascular volume and cardiac contractility [9].

During laparoscopic surgery, peak inspiratory pressure increases by 19% in the Trendelenburg position and 32% during insufflations [10]. Neonates, infant, and children who have a low FRC and high closing capacity and oxygen consumption are more prone to develop hypoxemia following increased intra-abdominal pressure and so increased pulmonary vascular resistance due to this hypoxemia [11].

The incidence of congenital tracheal stenosis (CTS) in infants with CHD has been reported to be 2.5% [12, 13]. Three types of CTS are described; segmental, funnel-shaped, and generalized hypoplasias [14]. The adequate ventilatory strategy is to prolong the inspiratory time but to overcome the increased resistance and achieve adequate lung expansion, and the respiratory rate should be kept at a low rate to allow adequate time for expiration and to prevent gas trapping [15]. In addition to positive pressure ventilation, high-frequency jet ventilation and extracorporeal membrane oxygenation have been used for patients undergoing surgical repair of tracheal lesions [12].

## Conclusions

In summary, anesthetic care of patients with tracheobronchial abnormalities demands particular attention. The identification of cardiovascular comorbidities is the most challenging task, and meticulous preoperative evaluation of heart defects should be warranted. However, anesthetists should be aware of the fact that the management of these patients should discuss all the multisystemic aspects of this complex syndrome. Bronchial branching malformation like BB with or without stenosis is an important differential diagnosis in newborn presenting with respiratory distress.
